# Variations in Complete Blood Counts in Patients With Non-hematological Malignancies: Insights From a Retrospective Hospital-Based Study

**DOI:** 10.7759/cureus.81257

**Published:** 2025-03-26

**Authors:** Garima Anandani, Vaishali Bhankhodia, Sagar Dholariya

**Affiliations:** 1 Pathology, All India Institute of Medical Sciences, Rajkot, Rajkot, IND; 2 Biochemistry, All India Institute of Medical Sciences, Rajkot, Rajkot, IND

**Keywords:** anemia, hematological changes, leukocytosis, non-hematological malignancies, thrombocytosis

## Abstract

Introduction: Malignancy ranks among the leading causes of mortality in both developed and developing nations. An abnormal hematological profile may serve as the initial indication of various non-hematological malignancies or solid tumors.

Aims and objectives: This research aims to investigate the spectrum of hematological alterations in individuals diagnosed with non-hematological cancers, evaluate their prevalence, and determine the relationship between complete blood count results and specific types of malignancies at a tertiary care facility in Gujarat.

Materials and methods: We compiled the retrospective data of 135 cases diagnosed with non-hematological malignancies through histopathological examination, all of whom had a CBC report and sought consultation at our hospital between January 2022 and December 2024. The CBC reports for all included cases were thoroughly analyzed. Statistical analysis was performed using the Statistical Package for Social Sciences version 28.0 (IBM Corp., Armonk, NY). Continuous variables were expressed as mean ± standard deviation, while categorical variables were represented as counts and percentages.

Results: There was a male preponderance amongst cases with head and neck (HN) cancer as the most common non-hematological malignancy. Anemia was observed as an important hematological finding predominantly in female patients. Leucocytosis, neutrophilia, eosinophilia, and thrombocytosis were also identified in some cases, especially in those who were non-anemic. HN cancer and malignancies of the female genital tract demonstrated significant anemia, lung tumors had markedly elevated red blood cell counts, and breast cancer patients were associated with thrombocytosis.

Conclusion: The current research demonstrated a definite correlation between hematological manifestations, such as anemia, leucocytosis, and thrombocytosis, and various non-hematological malignancies. Early detection of these conditions during disease progression can significantly reduce associated morbidity and mortality, ultimately contributing to improved patient care.

## Introduction

Malignancy is one of the primary causes of death in both developed and developing countries. Various types of cancer present a diverse array of symptoms. Often, an abnormal hematological profile may be the first sign of several non-hematological malignancies. These hematological irregularities can present as anemia, polycythemia, leukocytosis, thrombocytosis, monocytosis, and eosinophilia. The mechanisms underlying these hematological disorders are characterized by the release of various humoral factors from both cancer cells and normal splenic cells in individuals with cancer. Substances such as granulocyte-macrophage colony-stimulating factor (GM-CSF), interleukin (IL)-6, IL-1, and tumor necrosis factor (TNF) have been associated with these conditions. These tumor-derived factors can either stimulate or suppress normal hematopoiesis, produce antibodies that cross-react with receptors on specific cell types, or directly infiltrate the bone marrow [1]. 

Anemia is a prevalent condition observed in patients with cancer, and cancer is among the chronic illnesses most commonly associated with anemia [1, 2]. The presence of anemia may stem from the malignancy itself, acute or chronic blood loss, hemolysis, the marrow-suppressive effects of treatment, or as a consequence of chronic disease [3]. An increase in the white blood cell (WBC) count is also observed in several forms of non-hematological cancers, particularly among individuals diagnosed with lung cancer [4]. Eosinophilia and monocytosis, which can serve as early indicators of malignancy, are frequently encountered and present in a majority of patients. Malignancy is a key contributor to secondary or reactive thrombocytosis, which has been documented in numerous solid tumors [3].

Aims and objectives

Aims 

This research aims to investigate the range of hematological changes in patients with non-hematological malignancies, assess their frequency, and establish a correlation between complete blood count (CBC) findings and particular malignancy types at a tertiary care center in Gujarat.

Objectives

(1) To study the hematological variations in non-hematological malignancies at a tertiary care center in Gujarat; (2) to identify the frequencies of distinct non-hematological malignancies and correlate them with changes in complete blood count. 

## Materials and methods

This retrospective cross-sectional record-based study was conducted in the Department of Pathology at a tertiary care center in Gujarat. This is a retrospective record-based study approved by the Institutional Ethics Committee (IEC Approval Number- IEC/AIIMS/RAJKOT/5th/ER/16).

Individuals of any age and sex, who were newly diagnosed on biopsy to have a non-hematological malignancy and untreated, having a CBC report were included in the study. We excluded cases who had hematological malignancies; and those cases of non-hematological malignancies whose CBC report was not available in the hospital information system (HIS).

We compiled the retrospective record-based data of 135 patients who visited our hospital for consultation from January 2022 to December 2024. The histopathological slides were retrieved and re-examined for all the included cases for validation and confirmation of the diagnosis in HIS. The CBC reports of all these included cases were analyzed. The abnormalities in CBC findings according to the normal reference ranges, including changes in hemoglobin (Hb), red blood cell (RBC) indices, WBC count, and platelet counts were studied in various non-hematological malignancies [5].

Anemia represents a significant public health challenge worldwide, impacting individuals across all age groups in both developing and developed nations. The World Health Organization (WHO) defines anemia as having Hb levels below 12.0 g/dL for women and below 13.0 g/dL for men [6]. According to the WHO, anemia was graded as mild anemia if Hb levels were between 11.0 and 11.9 g/dL, moderate anemia if Hb levels were within the range of 8.0 to 10.9 g/dL, and severe anemia if Hb was less than 8.0 g/dL [7].

Statistical analysis

The statistical analysis was conducted to assess hematological variations and their associations with non-hematological malignancies. Continuous variables were evaluated for normality using the Shapiro-Wilk test. Parametric data (mean ± standard deviation (SD)) were analyzed with one-way ANOVA, while non-parametric data (median / inter-quartile range (IQR)) were analyzed using the Kruskal-Wallis test. Categorical variables (e.g., anemia prevalence) were presented as numbers and percentages (%) and compared using chi-square or Fisher’s exact test, as appropriate. All analyses were performed by using SPSS v. 28.0 (IBM Corp., Armonk, NY) and Microsoft Excel (Microsft Corp., Redmond, WA). A p-value of less than 0.05 was considered significant.

## Results

Among the 135 patients with non-hematological malignancies, the majority were middle-aged (mean age: 51.0 ± 13.0 years), with a pronounced male predominance (72.6%, n=98; female: 27.4%, n=37). The age distribution showed 48.1% (n=65) in the 25-50 age group and 49.6% (n=67) above 50; notably, only three patients (2.2%) were ≤25 years old, suggesting rare incidence or underreporting of malignancies in younger populations. The pronounced gender disparity (male-to-female ratio: 2.6:1) may reflect regional risk factors like tobacco use, occupational exposures, or healthcare access patterns in Gujarat (Tables 1-2).

**Table 1:** Demographic and Clinical Characteristics of Patients With Non-hematological Malignancies

Variable	Category	Frequency (n)	Percentage (%)	Mean ± SD
Age (years)	-	-	-	51.0 ± 13.0
Sex	Male	98	72.6%	-
	Female	37	27.4%	-
Site of Malignancy	Head and neck	82	60.7%	-
	Female genital tract	17	12.6%	-
	Lung	16	11.9%	-
	Breast	6	4.4%	-
	Skin	5	3.7%	-
	Bone	5	3.7%	-
	Others (Soft tissue, Neural, Prostate)	4	3%	-

**Table 2:** Distribution of Patients With Non-hematological Malignancies According to Age

Age Group	Males	Females	Total
≤25 years	2 (2.04%)	1 (2.70%)	3 (2.22%)
>25 to ≤50 years	43 (43.88%)	22 (59.46%)	65 (48.15%)
>50 to ≤60 years	29 (29.59%)	7 (18.92%)	36 (26.67%)
>60 years	24 (24.49%)	7 (18.92%)	31 (22.96%)
Total	98 (72.59%)	37 (27.41%)	135 (100%)

Head and neck (HN) malignancies were the most common overall (60.7%), with a strong male predominance (72.4%), particularly oral cavity cancers (58.2% of HN cases), likely linked to tobacco use. In females, female genital tract (FGT) cancers (45.9%) and breast cancers (16.2%) were most prevalent. Breast cancers (4.4%) were underrepresented compared to global trends, potentially due to referral biases or population-specific etiologies. Less frequent malignancies included lung (11.9%), skin (3.7%), bone (3.7%), and rare soft tissue/neural/prostate cancers (3%). Males exhibited higher rates of lung (15.3%) and skin cancers (5.1%), while female malignancies were concentrated in reproductive organs (uterus: 21.6%, cervix: 16.2%). Notably, prostate, thyroid, and neural cancers were rare (<1-2.7%). These patterns highlight gender-specific oncological trends, emphasizing the need for targeted screening and regional risk factor mitigation (e.g., tobacco use for HNC and human papillomavirus (HPV) for cervical malignancies) (Table 3).

**Table 3:** Gender-Specific Distribution of Malignancy Types Among Patients With Non-hematological Malignancies

Malignancy Type	Males (n=98)	Females (n=37)	Total (n=135)
Head and Neck	71 (72.4%)	11 (29.7%)	82 (60.7%)
Oral cavity	57 (58.2%)	8 (21.6%)	65 (48.1%)
Larynx	4 (4.1%)	0 (0%)	4 (3.0%)
Face	7 (7.1%)	2 (5.4%)	9 (6.7%)
PNS	1 (1.0%)	0 (0%)	1 (0.7%)
Salivary gland	1 (1.0%)	0 (0%)	1 (0.7%)
Thyroid	0 (0%)	1 (2.7%)	1 (0.7%)
Lymph node	1 (1.0%)	0 (0%)	1 (0.7%)
Female genital tract	-	17 (45.9%)	17 (12.6%)
Uterus	-	8 (21.6%)	8 (5.9%)
Cervix	-	6 (16.2%)	6 (4.4%)
Vault	-	2 (5.4%)	2 (1.5%)
Ovary	-	1 (2.7%)	1 (0.7%)
Lung	15 (15.3%)	1 (2.7%)	16 (11.9%)
Breast	0 (0%)	6 (16.2%)	6 (4.4%)
Skin	5 (5.1%)	0 (0%)	5 (3.7%)
Bone	4 (4.1%)	1 (2.7%)	5 (3.7%)
Soft tissue	1 (1.0%)	1 (2.7%)	2 (1.5%)
Neural	1 (1.0%)	0 (0%)	1 (0.7%)
Prostate	1 (1.0%)	-	1 (0.7%)
Total	98 (72.59%)	37 (27.41%)	135 (100%)

The hematological analysis revealed significant deviations from standard reference ranges. Anemia was prevalent, with mean hemoglobin (Hb) levels of 11.43 ± 2.69 g/dL and packed cell volume (PCV) at 35.78 ± 7.06%, falling below normal ranges for both genders. RBC indices indicated microcytic hypochromic anemia as mean corpuscular volume (MCV) was 77.46 ± 9.36 fL and mean corpuscular Hb (MCH) was 24.90 pg, likely linked to iron deficiency or chronic disease. Elevated red cell distribution width-coefficient of variance (RDW-CV) (15.44 ± 2.68%) suggested marked variability in RBC size. Platelet counts (median: 288 ×10³/µL) and leukocyte parameters (WBC: median 8.57 ×10³/µL) remained within normal limits. Neutrophils dominated the differential (65.72 ± 11.78%), with absolute neutrophil counts (ANC) (median 5.55 ×10³/µL) at the upper reference limit, hinting at underlying inflammation. Lymphocyte percentages (median 23.40%) were borderline low, potentially reflecting immune suppression. Eosinophil and basophil counts were minimally elevated but within normal ranges. These findings highlight a pattern of anemia and thrombocytosis in some patients, emphasizing the need for targeted monitoring of malignancy-associated hematological changes (Table 4).

**Table 4:** Hematological Profile of Patients With Non-hematological Malignancies RBC: red blood cell; PCV: packed cell volume; Hb: hemoglobin; MCV: mean corpuscular volume; MCH: mean corpuscular hemoglobin; MCHC: mean corpuscular hemoglobins concentration; RDW-CV: red cell distribution width: coefficient of variance; WBC: white blood cell; ANC: absolute neutrophil count; ALC: absolute lymphocyte count; AMC: absolute monocyte count; AEC: absolute eosinophil count; ABC: absolute basophil count; PLT: platelet * Median (IQR)

Parameter	Mean ± SD or Median (IQR) (Q1, Q3)*	Reference Range
RBC count (10⁶/µL)	4.59 ± 0.80	Male: 4.5-5.9, Female: 4.0-5.2
PCV (%)	35.78 ± 7.06	Male: 40-54, Female: 36-48
Hb (g/dL)	11.43 ± 2.69	Male: 13.5-17.5, Female: 12-15.5
MCV (fL)	77.46 ± 9.36	80-100
MCH (pg)	24.9 (21.5, 27.5)*	27-31
MCHC (g/dL)	31.62 ± 2.16	32-36
RDW CV (%)	15.44 ± 2.68	11-15
WBC Count (×10³/µL)	8.57 (6.58, 9.57)*	4-11
Neutrophils (%)	65.72 ± 11.78	40-70
Lymphocytes (%)	23.40 (16.7,30.1)*	20-40
Monocytes (%)	7.40 (5.6, 9.2)*	2-10
Eosinophils (%)	1.80 (0.8, 2.8)*	0-6
Basophils (%)	0.40 (0.55,0.25)*	0-2
ANC (×10³/µL)	5.55 (4.23, 6.87)*	2-7
ALC (×10³/µL)	1.90 (1.42, 2.38)*	1-4
AMC (×10³/µL)	0.64 (0.45,0.82)*	0.2-1
AEC (×10³/µL)	0.14 (0.06, 0.22)*	0-0.5
ABC (×10³/µL)	0.04 (0.02, 0.05)*	0-0.2
PLT (×10³/µL)	288.0 (219.0, 357.0)*	150-450

The study identified a high burden of anemia among patients with non-hematological malignancies, affecting 63.7% of the cohort (86/135). Females exhibited a notably higher prevalence (67.6%, 25/37) compared to males (62.2%, 61/98), despite the stricter diagnostic threshold for anemia in females (Hb <12 g/dL vs. Hb <13 g/dL for males). This disparity may reflect biological differences (e.g., menstruation-related iron loss), malignancy-specific impacts (e.g., gynecological cancers), or systemic effects of chronic disease. The overall prevalence underscores anemia as a critical comorbidity in cancer care, necessitating routine screening and targeted interventions to mitigate its effects on treatment outcomes and quality of life. These findings highlight the urgent need for integrated hematological monitoring in oncology protocols (Table 5). 

**Table 5:** Gender-Specific Prevalence of Anemia Among Patients With Non-hematological Malignancies Hb: hemoglobin

Sex	Criteria for anemia	Total cases	Anemic cases	Prevalence (%)
Male	Hb < 13 g/dl	98	61	62.2%
Female	Hb < 12 g/dl	37	25	67.6%
Total	-	135	86	63.7%

The analysis of anemia severity among patients with non-hematological malignancies revealed that moderate anemia (Hb 8.0-10.9 g/dL) was the most prevalent, affecting 60.5% of cases (n=52), underscoring chronic inflammation or malnutrition often linked to cancer progression. Severe anemia (Hb <8.0 g/dL) followed, impacting 25.5% of patients (n=22), a critical finding that highlights the urgency for clinical interventions such as transfusions or iron therapy. Only 14% of cases (n=12) were classified as mild (Hb 11-11.9 g/dL), suggesting that anemia in this population frequently progresses to advanced stages before detection. Collectively, these results emphasize the substantial burden of moderate-to-severe anemia in cancer care and the need for proactive hematological monitoring to improve patient outcomes (Table 6).

**Table 6:** Distribution of Anemia Severity Among Patients With Non-hematological Malignancies Hb: hemoglobin

Severity of anemia (Hb)	Number of cases (%)
Mild (Hb 11-11.9 g/dL)	12 (14.0%)
Moderate (Hb 8-10.9 g/dL)	52 (60.5%)
Severe (Hb <8.0 g/dL)	22 (25.5%)
Total	86 (100%)

In this study of non-hematological malignancies at a tertiary care center, hematological abnormalities were analyzed in relation to anemia. Percentages for anemia-related findings were calculated relative to the total anemic (n=86) and non-anemic (n=49) patients, while overall proportions reflected the entire cohort (n=135). Statistical significance (p<0.05) was determined using chi-square or Fisher’s exact tests. Neutrophilia (24.4% in anemic vs. 22.4% in non-anemic) and thrombocytosis (23.3% vs. 8.2%) showed significant associations with anemia (p=0.03 and p=0.02, respectively), implicating systemic inflammation and platelet hyperactivity as potential drivers. Conversely, leukocytosis (17.4% vs. 24.5%) and eosinophilia (4.7% vs. 10.2%) were more frequent in non-anemic patients but lacked statistical significance. Notably, leukopenia was absent, and thrombocytopenia was rare (1.5% overall). These findings underscore anemia’s strong ties to inflammatory and thrombotic pathways in non-hematological patients, advocating for prioritized monitoring of these parameters to improve clinical outcomes in malignancy care (Table 7).

**Table 7:** Prevalence and Associations of Hematological Abnormalities with Anemia in Patients With Non-hematological Malignancies p-value is calculated by chi-square or Fisher’s exact tests. *Fisher’s exact test was used wherever the number of cases are less than 5. ANC: absolute neutrophil count; ALC: absolute lymphocyte count; AMC: absolute monocyte count; AEC: absolute eosinophil count; ABC: absolute basophil count

Hematological abnormality	Anemia present: Cases (%)	Anemia absent: Cases (%)	Total cases (%)	p-value	Test used	Test value (chi-square or Fisher’s exact)
Leukocytosis (>11,000/µL)	15 (17.4%)	12 (24.5%)	27 (20.0%)	0.38	Chi-square test	0.77
Leukopenia (<4,000/µL)	0 (0.0%)	0 (0.0%)	0 (0.0%)	N/A	-	N/A
Neutrophilia (ANC >7,500/µL)	21 (24.4%)	11 (22.4%)	32 (23.7%)	0.03	Chi-square test	4.71
Eosinophilia (AEC ≥500/µL)	4 (4.7%)	5 (10.2%)	9 (6.7%)	0.30	Chi-square test	1.07
Lymphocytosis (ALC >4,000/µL)	4 (4.7%)	0 (0.0%)	4 (3.0%)*	0.15	Fisher’s exact test	0.15*
Monocytosis (AMC >1,000/µL)	12 (14.0%)	6 (12.2%)	18 (13.3%)	0.72	Chi-square test	0.13
Basophilia (ABC >100/µL)	2 (2.3%)	0 (0.0%)	2 (1.5%)*	0.51	Fisher’s exact test	0.51*
Thrombocytosis (>4 lakh/µL)	20 (23.3%)	4 (8.2%)	24 (17.8%)	0.02	Chi-square test	5.51
Thrombocytopenia (<1.5 lakh/µL)	1 (1.2%)	1 (2.0%)	2 (1.5%)*	1.00	Fisher’s exact test	1.00*

This study emphasizes notable hematological discrepancies among several non-hematological cancers. HN cancers and malignancies of the FGT demonstrated significant anemia, with Hb levels of 11.2 ± 2.9 g/dL and 10.9 ± 2.1 g/dL, respectively (p < 0.001). Lung tumors had markedly elevated RBC counts (5.01 ± 0.98 ×10⁶/µL, p = 0.03). Thrombocytosis was most pronounced in breast cancer, with a median platelet count of 386 ×10³/µL (p = 0.01), perhaps reflecting underlying inflammation or tumor-induced hematopoietic activation. Furthermore, mean corpuscular hemoglobin concentration (MCHC) and RDW-CV exhibited substantial intergroup differences (p < 0.05), indicating dysregulated erythropoiesis among malignancies (Tables 8-9).

**Table 8:** Comparative Parametric test data for Hematological Profiles Across Non-hematological Malignancies *p-value is significant. RBC: red blood cell; PCV: packed cell volume; Hb: hemoglobin; MCV: mean corpuscular volume; MCH: mean corpuscular hemoglobin; MCHC: mean corpuscular hemoglobin concentration; RDW-CV: red cell distribution width: coefficient of variance

Parametric data	Head and neck tumors	Female genital tract tumors	Lung tumors	Breast tumors	Bone tumors	Skin tumors	p-value	Test Statistic (F-value using one-way ANOVA test)
	Mean ± Standard deviation							
RBC count (10⁶/µL)	4.45 ± 0.78	4.34 ± 0.76	5.01 ± 0.98	4.35 ± 0.78	5.25 ± 0.64	4.28 ± 0.28	0.03*	4.56
PCV (%)	35.8 ± 7.2	34.2 ± 5.9	39.8 ± 7.4	32.2 ± 7.1	39.3 ± 4.3	31.3 ± 4.5	0.01*	5.23
Hb (g/dL)	11.2 ± 2.9	10.9 ± 2.1	12.8 ± 2.3	10.3 ± 2.2	13.1 ± 1.6	9.3 ± 1.9	<0.001*	9.87
MCV (fL)	77.8 ± 9.5	76.8 ± 9.1	75.6 ± 9.7	73.9 ± 12.3	76.6 ± 8.8	72.3 ± 8.9	0.12	1.89
MCH (pg)	24.6 ± 3.1	25.1 ± 3.3	24.1 ± 3.5	20.8 ± 3.2	24.2 ± 3.6	20.4 ± 2.9	0.02*	3.78
MCHC (g/dL)	31.6 ± 2.3	32.1 ± 2.0	31.5 ± 1.9	29.6 ± 2.1	31.6 ± 2.1	29.9 ± 1.7	0.004*	5.45
RDW CV (%)	15.6 ± 2.4	14.1 ± 4.2	14.3 ± 1.7	17.6 ± 1.2	14.6 ± 2.3	17.1 ± 4.1	0.01*	4.12

**Table 9:** Comparative Non-parametric Test Data for Hematological Profiles Across Non-hematological Malignancies *p-value is significant. WBC: white blood cell; ANC: absolute neutrophil count; ALC: absolute lymphocyte count; AMC: absolute monocyte count; AEC: absolute eosinophil count; ABC: absolute basophil count; PLT: platelet

Non- Parametric data	Head and Neck	Female genital tract	Lung	Breast	Bone	Skin	p-value	Test Statistic (H-value using Kruskal-Wallis test)
	Median (Range)							
WBC Count (×10³/µL)	7.9 (6.3-10.1)	8.7 (7.5-12.3)	9.4 (8.2-14.2)	10.1 (9.6-11.8)	7.5 (5.3-12.3)	7.3 (5.5-12.5)	0.32	4.56
Neutrophils (%)	64.0 (55.4-73.8)	65.8 (59.4-74.1)	68.0 (61.3-77.2)	71.3 (64.2-78.7)	66.7 (63.0-68.5)	58.6 (51.2-62.4)	0.04*	6.12
Lymphocytes (%)	24.4 (16.0-32.3)	23.4 (16.0-32.7)	20.5 (15.6-23.4)	20.1 (14.9-28.7)	22.4 (19.0-26.6)	27.6 (26.4-37.2)	0.18	3.45
Monocytes (%)	7.8 (5.9-9.8)	6.6 (5.1-7.6)	7.6 (5.9-9.6)	6.6 (5.4-7.4)	7.3 (4.8-11.3)	8.4 (8.2-11.6)	0.45	2.34
Eosinophils (%)	1.5 (0.4-3.0)	1.9 (0.5-3.4)	1.4 (0.5-2.4)	0.9 (0.6-1.7)	1.4 (0.6-9.2)	1.6 (1.4-4.0)	0.60	1.89
Basophils (%)	0.4 (0.2-0.7)	0.4 (0.2-0.5)	0.3 (0.2-0.4)	0.3 (0.2-0.6)	0.4 (0.3-0.5)	0.4 (0.2-0.4)	0.87	0.78
ANC (×10³/µL)	4.9 (3.4-6.9)	5.6 (4.3-8.2)	6.7 (5.3-8.6)	6.9 (6.0-9.4)	5.1 (3.4-8.3)	4.6 (3.2-6.4)	0.03*	5.89
ALC (×10³/µL)	1.9 (1.4-2.6)	2.1 (1.5-2.9)	2.0 (1.7-2.3)	2.4 (1.8-3.1)	1.9 (1.4-2.8)	2.0 (1.6-4.6)	0.09	4.23
AMC (×10³/µL)	0.6 (0.4-0.8)	0.5 (0.4-0.7)	0.8 (0.5-1.1)	0.6 (0.5-0.7)	0.4 (0.4-0.9)	0.6 (0.5-1.2)	0.21	3.12
AEC (×10³/µL)	0.1 (0.04-0.2)	0.1 (0.04-0.2)	0.1 (0.05-0.2)	0.1 (0.08-0.2)	0.1 (0.06-0.8)	0.1 (0.1-0.2)	0.75	1.45
ABC (×10³/µL)	0.04 (0.02-0.05)	0.03 (0.02-0.04)	0.03 (0.02-0.06)	0.04 (0.02-0.06)	0.04 (0.03-0.05)	0.03 (0.02-0.04)	0.50	2.01
Plt (×10³/µL)	248 (216-288)	286 (242-410)	337 (262-382)	386 (168-398)	330 (286-406)	292 (237-352)	0.01*	7.23

Neutrophilia was markedly increased in lung cancer (median 68%, p = 0.04), with a higher ANC (p = 0.03), indicating a systemic inflammatory response typically seen in pulmonary malignancies. Notwithstanding these disparities, the counts of monocytes, eosinophils, and basophils exhibited no significant variation among cancer types (p > 0.05), suggesting that these parameters may be less affected by tumor load or inflammatory processes in solid malignancies. Moreover, MCV, total WBC count, and lymphocyte percentages exhibited no significant intergroup variation (p > 0.05), indicating that these metrics may not be dependable hematological markers for differentiating between various cancers.

From a therapeutic standpoint, these findings emphasize the necessity for malignancy-specific hematological surveillance. The pronounced anemia noted in cutaneous and female genital tract malignancies may stem from persistent inflammation associated with disease, dietary inadequacies, or suppression of bone marrow function. Thrombocytosis observed in breast and lung malignancies is likely associated with tumor-induced cytokine release and heightened thrombopoietic activity, thereby increasing thrombotic risks in these patients. The neutrophilia noted in lung malignancies corresponds with previous findings indicating a persistent inflammatory microenvironment in lung malignancy, possibly influenced by cytokines such as G-CSF. These hematological changes highlight the necessity of thorough blood parameter analysis in oncology, facilitating improved risk assessment, prognostic evaluation, and customized treatment strategies.

## Discussion

Haematological disorders frequently occur in conjunction with non-haematological cancers. Notably, cancer-related anemia presents a considerable healthcare challenge, impacting approximately 75% of cancer patients throughout their illness. This condition diminishes their quality of life by inducing symptoms such as fatigue, lethargy, decreased appetite, and impaired concentration. Furthermore, anemia exacerbates cancer prognosis, prolongs treatment duration, and contributes significantly to morbidity among cancer patients, ultimately affecting survival rates [1].

Anemia in cancer patients can arise from both cancer-related and non-cancer-related factors. Non-cancer-related causes may include underlying comorbidities such as bleeding, hemolysis, nutritional deficiencies, hemoglobinopathies, and infections. In the context of cancer, anemia can be attributed to the direct effects of tumors or the side effects of treatments. Solid tumors can infiltrate the bone marrow, leading to the suppression of hematopoiesis, or they may activate the immune system, resulting in the production of cytokines such as IL-1, interferon-gamma, IL-6, and TNF-alpha. These cytokines can contribute to decreased Hb levels by promoting hemolysis, inhibiting erythropoiesis, and interfering with the release and synthesis of endogenous erythropoietin [1, 2, 8, 9]. Iron deficiency anemia (IDA) is frequently noted in cancer and is associated with advanced disease, close proximity to cancer therapy, and poor performance status in patients with solid tumors [10].

In our research, anemia emerged as a significant hematological observation in 63.7% of the cases with various non-hematological malignancies, which is similar to the studies done by various other authors [1, 3, 11, 12]. Ludwig et al. identified a prevalence of anemia amongst patients diagnosed with solid tumors to be 50.4%, while Almehmadi et al. reported it to be 44.1% in Saudi Arabian patients, Merlini et al. as 32%, and Steegmann et al. as 48.1 % [13-16]. In this study, anemia was more common in females, similar to Kifle et al., who reported 66% of such cases as females, with 36% of them in the 35-49 year age group [17].

Previous studies have also indicated that in non-haematological malignancies, anemia is more prevalent among female patients, along with older individuals [13, 18, 19].

This study classified anemia into three categories based on severity, with moderate anemia being the most frequent. most commonly identified to be IDA and anemia of chronic disease, consistent with the results reported by Dandavate et al [1]. According to the findings of Muthanna et al., 56.6 % of such cases exhibited mild anemia, while 34.2 %exhibited moderate anemia, and 9.1 % severe anemia [19]. Ludwig et al. indicated that 29.7 % of patients with solid tumors were affected by moderate to severe anemia [13].

According to Dandavate et al. microcytic morphology was predominant, accounting for 55.88%, whereas macrocytic morphology was observed in only 8.8% of the cases [1]. Also in the research conducted by Kifle et al., it was found that 47.4% of the anemia cases were classified as microcytic, while 50.5% were identified as normocytic [17]. In contrast, Muthanna et al. identified that the predominant forms of anemia were normocytic normochromic, accounting for 75%, and macrocytic polychromic represented 17.5% [19]. Schwartz reported analogous results, indicating that predominantly normocytic normochromic anemia was present among patients with cancer [20].

In our research, HN cancer emerged as the most prevalent, followed by FGT cancers, and both were associated with anemia. A study carried out in Sudan identified breast cancer (23.1%), colorectal/anorectal cancer (18.7%), HN cancer (17.6%), and FGT cancer (14.9%) as the predominant cancer types. Notably, anemia was observed in 50.3% of patients diagnosed with malignant tumors. [18]. Kifle et al. reported that gynecologic cancers were the most common, followed by nasopharyngeal, colorectal, soft tissue sarcoma, and HN cancers [17]. Xu et al. reported breast cancer to be the most prevalent, followed by lung, colorectal, ovarian, and gastric cancer [21]. 

Breast cancer patients exhibited the highest prevalence of anemia (41.1%) in Muthanna et al., in contrast to the study by Ludwig et al., showing the highest prevalence of anemia in lung cancer and gynecological cancers [19, 22]. In Abuidris et al., anemia was most prevalent in prostate (82.9%) and urinary system (75.0%) cancer, which might be due to hematuria [18].

In Ethiopia, 23% of such cases had anemia, with the highest occurrence in gynecologic (37.7%) and colorectal (26.7%) cancers [17]. In the United States, the prevalence of anemia ranged from 26% in colorectal cancer to 59% in ovarian cancer [21]. Beale et al. identified colorectal cancer to be most commonly associated with anemia in the UK [23].

In this study, leukocytosis was identified in 20% and neutrophilia in 23.7% of the cases, similar to various other studies [3, 24, 25]. Granger et al. showed that leucocytosis generally had neutrophilia and radiographic findings, suggestive of metastatic disease [26]. However, Shoenfeld et al. reported monocytosis to be 25% among such patients, unlike our study [27]. Increased platelet count was observed in 17.8% of the cases in the current study, supporting the results reported by other researchers [3, 28-30]. This type of study is the first of its kind in the Indian state of Gujarat, where a range of solid tumors are correlated with the hematological changes. However, the limitations of this study were a small sample size and possible selection bias as it was a single-centre study. 

## Conclusions

There is a significant correlation between hematological manifestations, such as anemia, leucocytosis (including neutrophilia, eosinophilia, and monocytosis), and thrombocytosis with various non-hematological cancers. A notable prevalence of anemia was observed in newly diagnosed, untreated non-hematological cancer patients, with iron deficiency anemia being the most frequently encountered type, followed by anemia of chronic disease. Timely diagnosis and monitoring of hematological irregularities can help mitigate associated morbidity and mortality, thereby facilitating appropriate treatment and preserving a high quality of life.
